# Acknowledgement to Reviewers of *Nanomaterials* in 2016

**DOI:** 10.3390/nano7010014

**Published:** 2017-01-12

**Authors:** Nanomaterials Editorial Office

**Affiliations:** MDPI AG, St. Alban-Anlage 66, 4052 Basel, Switzerland; nanomaterials@mdpi.com

The editors of *Nanomaterials* would like to express their sincere gratitude to the following reviewers for assessing manuscripts in 2016.

We greatly appreciate the contribution of expert reviewers, which is crucial to the journal’s editorial process. We aim to recognize reviewer contributions through several mechanisms, of which the annual publication of reviewer names is one. Reviewers receive a voucher entitling them to a discount on their next MDPI publication and can download a certificate of recognition directly from our submission system. Additionally, reviewers can sign up to the service Publons (https://publons.com) to receive recognition. Of course, in these initiatives we are careful not to compromise reviewer confidentiality. Many reviewers see their work as a voluntary and often unseen part of their role as researchers. We are grateful to the time reviewers donate to our journals and the contribution they make.

If you are interested in becoming a reviewer for *Nanomaterials*, see the link at the bottom of the webpage http://www.mdpi.com/reviewers.

The following reviewed for *Nanomaterials* in 2016:
Abidi, NoureddineHogarth, GraemePiletsky, SergeyAfarinkia, KamyarHolappa, LauriPilo-Pais, M.Agrawal, Kumar VaroonHolden, PatriciaPinto, ArturAili, DavidHong, YongtaekPinto, RicardoAlbergaria, José TomásHoogenboom, JacobPires, Ricardo A.Alcoutlabi, MatazHöppener, StephaniePirola, CarloAldakov, DmitryHorgnies, MatthieuPita, MarcosAlexiou, ChristophHorvat, JosipPitard, BrunoAlexis, FrankHsu, Wei-HsuanPodlaha-Murphy, ElizabethAlonsoa, FranciscoHu, AnmingPolavarapu, LakshminarayanaAmarandei, GeorgeHu, ShanghsiuPolitano, OlivierAndrews, MarkHuang, Bohr-RanPollmann, KatrinAnsón-Casaos, AlejandroHuang, Chin-PaoPowell, AlexanderAntaryami, MohantaHuang, QijinPozuelo, JavierAo, ZhiminHuang, XiaohuaPralle, ArndAoki, TakeshiHuh, Yong-MinPrassl, RuthArango, AlexiHung, Kuo-YungPuiggalí, JordiArdanuy, MónicaHung, Wei HsuanPulido, GerardoArgyropoulos, ChristosHwang, JunghoPum, DietmarAriga, KatsuhikoHwang, SeongpilQuattrocelli, MattiaArmentano, IlariaIchchou, MnQuevedo, ManuelArscott, SteveIjiro, KuniharuRack, Philip D.Ashraf, ShahidIqbal, ZafarRadenovic, AleksandraAuth, ThorstenIreland, TerryRadovanovic, PavleAvgeropoulos, ApostolosIshihara, KazuyukiRahman, MasudurAzamat, JafarIshikawa, RyoRamiasa, MelanieAzuma, KazuoIslam, Md MahbubulRana, DipakBaas, Arnold S.Island, Joshua O.Rana, SubinoyBabu, RameshItaliani, PaolaRandall, ChristopherBagby, MichaelIto, SeigoRatova, MarinaBall, RichardIvanov, Dimitri A.Raucher, DrazenBan, MasahitoIvask, AngelaRawls, Henry R.Bang, Jun-HwanJacopin, GwénoléRazavi, PedramBanobre, ManuelJaenicke, StephanRebollar, EstherBaptista, PedroJank, Michael Peter MaxRebrov, EvgenyBarbier, AntoineJayamohan, HarikrishnanReddy, M. V.Barbillon, GrégoryJayan, B. ReejaReimhult, ErikBarranco, AngelJayant, Rahul DevRescignano, NicolettaBarron, Andrew R.Jelliss, Paul A.Rhee, Shi-WooBaşağaoğlu, HakanJensen, BenjaminRibis, JoelBaskoutas, SotiriosJeong, Yong YeonRicart, SusagnaBauer, FranqoisJeong, YoungjinRichard, LiptakBayer, Ilker S.Jerby, EliRinaldi-Montes, NataliaBeall, Gary W.Jeyabharathi, ChinnaiahRistori, SandraBeattie, James K.Jiang, JiangRizzi, Gian AndreaBeer, RossJiang, JunhuaRizzolio, FlavioBenelli, GiovanniJiang, ZhangRobert, DidierBerenguer-Murcia, ÁngelJiu, JintingRobinson, J. PaulBerenjian, AydinJoglekar, MadhuraRoduner, EmilBergamaschi, EnricoJokerst, JesseRolfe, PeterBerrier, EliseJoner, ErikRolinski, OlafBertocci, FrancescoJudy, JonathanRoper, D. KeithBertran, JoanJung, Woo-SikRosenkranz, AndreasBeygui, Ramin E.Jung, YeonwoongRuffino, FrancescoBhatnagar, AmitKageshima, HiroyukiRuppalt, Laura B.Bini, FabianoKah, MelanieRurali, RiccardoBirch, DavidKaittanis, CharalambosSa, JacintoBirkett, MartinKallio, TanjaSaafi, MohamedBloh, Jonathan Z.Kamada, KaiSada, KazukiBludov, YuKanaev, AndreiSaghafi, H.Boccaccini, AldoKaneko, TatsuoSalehi-Khojin, AminBohayra Mortazavi, BohayraKang, Hyun WookSalerno, MarcoBoldizar, AntalKang, PilgyuSalieri, BeatriceBolivar, JuanKantartzis, Nikolaos V.Sanchez, M.Bolotov, LeonidKatapodis, PetrosSanchez-Cortes, SantiagoBolton, KimKatsumata, HideyukiSandri, GiuseppinaBonferoni, Maria CristinaKawamura, AkifumiSantillan-Jimenez, EduardoBorges, João P. M. R.Kechrakos, DimitrisSaris, PerBoughton, BerinKelder, Erik M.Sarswat, Prashant K.Bouyer, FredericKemmegne-Mbouguen, Justin ClaudeSasaki, KotaroBradley, SiobhanKevadiya, BhaveshSasson, YoelBrendlé, JocelyneKhaleque, AbdulSato, SoshiBrennan, MareeKhang, DongwooScheicher, Ralph H.Brueck, Steve R. J.Kichambare, PadmakarSchmuki, PatrikBrunner, Andreas J.Kiesewetter, Dale O.Schmutz, MarcBuccheri, Maria-AntoniettaKim, Do KyungSchönherr, HolgerBussolati, OvidioKim, Hak-YongSeifert, GotthardByun, Hong-SikKim, Hyug-HanSemprini, LewisCai, QianKim, Jong HoSenyuk, BohdanCalabrese, GabrieleKim, Jung HoSerra, RafaelCalvo-Guirado, José LuisKim, Kwang SooSevostianov, IgorCamposeo, AndreaKim, Kyo-SeonSfyris, DimitrisCannio, MariaKim, Sang JaeShanmugam, SangarajuCao, DaweiKim, SangsikShapter, Joe G.Carniato, FabioKim, Seong WooSharifi, TivaCarreau, PierreKirby, JasonSharma, AdityaCaruntu, DanielaKirstein, StefanSharma, Virender K.Caruthers, Marvin H.Klaper, RebeccaShehata, NaderCasettari, LucaKlein, EricShen, Ji-LinCasey, AlanKloc, ChristianShih, Yang-HsinCassagnau, P.Klotzkin, DavidShimoi, NorihiroCassidy, JohnKnauth, PhilippeShin, Su RyonCastaño, PedroKnetsch, Menno L. W.Shin, Weon GyuCatalano, StefaniaKo, Dong KyunShiraishi, YasuhiroCatalina-Gabriela, SanporeanKo, Seung HwanShrestha, Lok KumarCavaliere, SaraKoeller, ManfredShukla, SourabhCelzard, AlainKoivisto, JoonasSingh, VijayCha, Sang-HoKoos, Antal A.Sisti, LauraChai, YangKoshimizu, MasanoriSkotadis, EvangelosChan, K. S.Koskinen, JariSmejkalova, DanielaChanana, MunishKourkoumelis, NikolaosSmith, Andrew M.Chang, Chih-YuKuang, HuihuiSnee, PrestonChang, SehoonKubař, TomasSnyder, JoshuaChang, Yu-ChengKubo, TakanoriSnyder, NicoleCharwat, VerenaKulcinski, GeraldSobolev, KonstantinChase, George G.Kuttner, ChristianSohn, YoungkuChatterton, Nicholas PaulKuzum, DuyguSokolov, IgorChaurand, PierreLai, James J.Solla-Gullón, JoséChen, BinlingLak, AidinSon, Seung UkChen, Chuh-YungLan, TianSonar, PrashantChen, ChunhuiLança, CarmoSong, JinhuiChen, JiaoLarraneta Landa, EnekoSong, KenanChen, Min-HueyLaurencin, CatoSoucy, GervaisChen, Ruei-TangLee, Chang-SeopSoukiassian, PatrickChen, Yang-FangLee, Chia-HungSousa, Célia T.Cheng, Fong-YuLee, DaehoSpitzer, DenisCheng, Kong-WeiLee, Geun-HyoungSrinivasan, MadapusiCheng, MarkLee, JiyeSrivastava, AshokCheng, Yang-TseLee, KwangyeolSrivastava, VarshaCheong, Yuen-KiLee, Sang-WhaStamatas, GeorgiosChipara, DorinaLee, WeiStanca, Sarmiza ElenaChithrani, Basnagge DevikaLee, Y.Stefanidou, MariaChiu, Po-WenLee, Yong-KyuStefano, Luca DeCho, HyunLefèvre, GrégoryStephan, HolgerCho, MichaelLevchenko, IgorStout, David A.Cho, So-HyeLi, JiantongStrano, MichaelChoi, Hyoung JinLi, JianyangStratakis, ManolisChoi, Jeong WooLi, JinghaoSubramaniam, PrasadChoi, JoshuaLi, Mei-ChunSuk, Jung SooChoi, SamjinLiagre, BertrandSumboja, AfriyantiChoudhary, NitinLiao, Jiunn-DerSun, An-ChengChu, BenjaminLin, YiSun, ChenghuaChu, Ching-LiangLin, Yu-ShenSun, XiaoleiChubar, NataliaLinares, MathieuSunasee, RajeshChueh, Yu-LunLing, Yong-ChienSzczepanik, BeataChunqi, QianLiu, BinTabe, ShahramClift, MartinLiu, HaipengTaboada, PabloClogston, Jeffrey D.LIU, HongweiTae, Heung-SikCole, Matthew T.Liu, JuewenTakeoka, ShinjiCollins, MauriceLiu, Qing-XiaTan, Chee-KeongColombo, MiriamLiu, RenTanaka, ManabuConstantoudis, VassiliosLiu, ZhuTasis, DimitriosCornell, BradfordLoh, KennethTavares, Carlos J.Costante, GiuseppeLongoni, GianlucaTchernycheva, MariaCragg, Peter J.Lopez, ReneTedbury, PhilipCremar, Lee DanielLópez-Lorente, Ángela I.Tejeda, AlexanderCunha, AngelaLopez-Ortega, AlbertoTerakawa, MitsuhiroDa Costa, Ana M. RosaLu, Ming-YenTeran, Francisco J.Das, NarottamLu, Peter J.Terry, IanDauskardt, Reinhold H.Luciani, PaolaTeschome, BezuDavies, Gemma-LouiseLuong, Dzung DinhThomson, AndrewDavis, Daniel C.Luzi, FrancescaThon, SusannaDe Aberasturi, Dorleta JiménezLyubchenko, Yuri L.Thünemann, AndreasDe Giglio, ElviraMa, HongyanThygesen, Kristian SommerDe Lacey, Antonio LópezMa, ShuangTiggelaar, Roland M.De Paiva Martins, Rodrigo FerrãoMacdonald, ThomasTiller, Joerg C.Del Pino, PabloMackowski, SebastianTilley, RichardDelville, Marie-HélèneMajumdar, SoumyajitTomitaka, AsahiDeria, PravasMalakooti, Mohammad H.Tomohiro, YokozekiDesideri, AlessandroMalik, Muhammad ArifTorreggiani, ArmidaDevaux, JacquesMalik, Muhammad AzadToupance, ThierryDeVries, K. LawrenceMallavia, RicardoTownley, Helen E.Dewitte, HeleenMalucelli, GiulioTrapalis, Christos C.Dey, SonalMaly, Kenneth E.Tricoli, AntonioDhakshinamoorthy, AmarajothiMani, VeerappanTrimmel, GregorDharmadasa, Imyhamy MudiyMannino, SaverioTrohidou, KalliopiDi Bartolomeo, AntonioManthina, VenkataTroughton, JoelDi Mauro, AlessandroMao, ChengdeTruong Phuoc, NghiaDi Palma, LucaMao, HanbinTsai, Chih-HungDi Stefano, AntonioMarbán, GregorioTsapis, NicolasDiamanti, Maria VittoriaMarder, Michael P.Tseng, I-HsiangDíez Barra, EnriqueMarks, Haley L.Tucker, NickDikin, Dmitriy A.Martinez, Julio A.Tulliani, Jean-MarcDinku, Abay GadisaMartinez-Manez, RamonTung, Chih-KuanDong, HongMartino, SabataUbertini, FilippoDopp, ElkeMartin-Ramos, PabloUm, Soong HoDorney, JenniferMatros, AndreaUnnithan, Ranjith RajasekharanDu, ShangfengMatten, Sharlene R.Valladares, Luis De Los SantosDu, XuMatteucci, ClaudiaVan Dyke, Mark E.Dunne, NicholasMatthias, LeinVankayala, RavirajDuponchel, LudovicMcPeak, KevinVarlamov, SergeyDurupthy, OlivierMeiners, SilkeVaseashta, Ashok K.Dutta, Pradip K.Meléndez, EnriqueVázquez, EsterDykman, Lev A.Melin, FredericVega, VictorEbara, MitsuhiroMestres, Adrià GilVerbruggen, Sammy W.Ebong, AbasifrekeMestroni, LuisaVerma, Navin KumarEfstathopoulos, Efstathios P.Meyer, FlorentVigolo, BrigitteElaissari, AbdelhamidMiguel, GraçaVijver, MartinaElemans, HansMiklavcic, StanVillalonga, ReynaldoElimelech, MenachemMing, Goh XiaoVivero-Escoto, Juan LuisEpifani, MauroMishra, Yogendra KumarVlček, Antonin, Jr.Erdmann, KatiMishur, Robert J.Vo, Minh D.Fabris, LauraMisra, PrabhakarVoliani, ValerioFanelli, FiorenzaMiyajima, ShinsukeVolkov, A.Fang, JiyeMiyatake, TomohiroVygranenko, YuriFang, YeMohanta, AntaryamiWalton, S. PatrickFedel, MariangelaMohl, MelindaWang, Chen-HaoFelderhoff, MichaelMoineau-Chane-Ching, Kathleen I.Wang, DanlingFeng, Z. VivianMolinari, AlessandraWang, JialaiFernández Romero, Juan ManuelMomeni, KasraWang, LudaFerreira, Isabel M. M.Monari, AntonioWang, Paul C.Figueiredo, MargaridaMondal, KunalWang, PengfeiFilpponen, IlariMondloch, JoeWang, QunFiorilli, SoniaMoon, Jong-YunWang, XiFollain, NadègeMorris, MichaelWang, XiupengFornasiero, PaoloMubeen, SyedWang, YunxiaoFrancesco, Fabio DiMukherji, DebashisWang, ZhiqiangFrey, MargaretMüller, DanielWatanabe, ToshioFröhlich, EleonoreMurase, NorioWeber, KelaFrontera, PatriziaMyong, SeungWeigand, RosaFujimoto, KeijiNagaoka, HiroyukiWen, XiaomingFujisaki, YoshihideNakano, KazuhikoWeng, QunhongFurusawa, HiroyukiNath, ManashiWiemann, MartinGamage McEvoy, JoanneNeuman, KeirWright, MatthewGao, WenpeiNguyen, TuyenWu, QinglinGao, YangNguyen-Huu, NghiaWu, Ren-JangGarcía-Márquez, AlfonsoNikolelis, DimitrosWu, Shu-PaoGarcía-Martinez, Joaquín C.Nilges, TomWu, SiGavin, HazellNydén, MagnusWu, TaoGea, Oliveri ContiNypelo, TiinaWu, XiangfaGendron, DavidOchiai, TsuyoshiWujcik, EvanGervasini, AntonellaOgris, ManfredWycisk, RyszardGhafari, EhsanOh, IlwhanXi, WeixianGiannakas, ArisÖhlund, ThomasXia, TianGibson, ElizabethOhulchanskyy, Tymish Y.Xing, YuqianGiese, BerndOkamoto, MasamiXu, TingtingGiovagnoli, StefanoOlthof, SelinaYamamoto, AkiraGodinho, MariaOoyama, YousukeYamauchi, SatoshiGoh, Suat HongOrtelli, SimonaYan, HeGomes, A.Ortolani, LucaYan, LiangGómez-Polo, CristinaOtto, CeesYang, MinghuiGonzález, SergioOuerghi, AbdelkarimYannopoulos, Spyros N.Gonzalez-Pedro, VictoriaPalchoudhury, SoubantikaYe, LongGoodwin, Andrew P.Palma, MatteoYeh, Yin-TingGopalakrishnan, GopanPan, LiangYentekakis, Ioannis V.Gopalakrishnan, Sai GautamPancrazio, JosephYi, LongGoreham, ReneePannier, Angela K.Yin, QianGranet, RobertPanomsuwan, GasiditYoon, MinjoongGrasset, FabienPapassiopi, NymphodoraYounesi, RezaGregory, Richard L.Park, NoejungYoung, SimmyGuerrero, MiguelPark, Soo-JinYu, AiminGuo, ChongshenPark, Sung HaYu, ChengzhongGurramkonda, ChandrasekharPark, Tae JooYu, Hak KiGutruf, PhilippParsons, Jason G.Yu, MengxiaoHa, Don-HyungPartch, Richard E.Yu, TaekyungHadipour, AfshinPascu, Sofia I.Yuan, HeyangHagiwara, HidehisaPassaglia, ElisaYuba, EijiHammerling, UlrichPatel, AnantZalnezhad, ErfanHan, XufengPaulmurugan, RamasamyZandi, OmidHanaor, DorianPeck, DonghyunZanella, CaterinaHanemann, ThomasPeijnenburg, WillieZangari, GiovanniHarris, David T.Peijnenburg, Willie J. G. M.Zhang, ChuanfangHempel, UtePeral, JoséZhang, JianHenderson, W. MatthewPeralta-Videa, Josezhang, LaichangHenniges, UtePereira, LucianaZhao, BoxinHenry, MartinPereiro, RosarioZheng, FanHernández, Sandra C.Pérez-Gil, JesúsZhi, C.Heys, JeffreyPericàs, Miquel A.Zhou, YuanyuanHideyuki, MitomoPeters, Ruud J. B.Zhu, ChengzhouHill, MichaelPetkov, NikolayZhu, LiangHilleringmann, UlrichPetrovykh, Dmitri Y.Zimmerman, Jeramy D.Hinestroza, JuanPeukert, WolfgangZin, ValentinaHippler, RainerPicart, CatherineZolotoukhina, TatianaHirsch, ThomasPichot, Vincent



